# Wheat *Myo-*inositol phosphate synthase influences plant growth and stress responses via ethylene mediated signaling

**DOI:** 10.1038/s41598-020-67627-w

**Published:** 2020-07-01

**Authors:** Naveen Sharma, Chanderkant Chaudhary, Paramjit Khurana

**Affiliations:** Department of Plant Molecular Biology, University of Delhi South Campus, Benito Juarez Road, New Delhi, 110021 India

**Keywords:** Plant biotechnology, Plant stress responses

## Abstract

L-*myo*-inositol phosphate synthase (MIPS; EC 5.5.1.4) is involved in abiotic stress tolerance, however its disruption and overexpression has also been associated with enhanced tolerance to pathogens. The molecular mechanism underlying the role of *MIPS* in growth, immunity and abiotic stress tolerance remains uncharacterized. We explore the molecular mechanism of *MIPS* action during growth and heat stress conditions. We raised and characterized the *TaMIPS* over-expressing rice transgenics which showed a reduced reproductive potential. Transcriptome analysis of overexpression transgenics revealed the activation of ET/JA dependent immune response. Pull-down analysis revealed the interaction of TaMIPS-B with ethylene related proteins. Our results suggest an essential requirement of *MIPS* for mediating the ethylene response and regulate the growth. A model is proposed outlining how fine tuning of *MIPS* regulate growth and stress tolerance of the plant.

## Introduction

L-*myo*-inositol phosphate synthase (MIPS; EC 5.5.1.4) is the sole enzyme for *myo*-inositol synthesis, a molecule of paramount importance during cell wall biogenesis^1^, auxin storage^2^, phytic acid synthesis^3^ and oligosaccharides synthesis^4^. Presence of *MIPS* in prokaryotes and eukaryotes, suggests its early existence during the course of evolution^5^. MIPS protein sequence has been under extensive conservation with existence of identical amino-acid stretches in the protein sequence such as (NGSPQN) and (SKSNV)^6,7^ and differential regulation of its activity by phosphorylation at serine residue. Phosphorylation of (NGSPQN) motif increases the enzyme activity, while that of the central serine residue of (SKSNV) motif inhibits its activity^6^. MIPS exist as an enzyme complex which is catered distinctly in different tissues depending upon the specific needs^8^.

Ectopic expression of MIPS results in enhanced tolerance towards cold, drought and salt stress in several plants^9–15^. Overexpression of *MIPS* also results in enhanced tolerance towards stem nematodes in transgenic sweet potato^15^. Interestingly, mutation of *AtMIPS1* results in light intensity dependent programmed cell death (PCD) and enhanced basal immunity^16,17^. Transcriptome analysis of *Atmips1* short-day and long-day grown plants reveals the up-regulation of defense related genes along with genes involved in salt stress and ozone stress^17^. Loss of function approach was used in different organism to reduce the expression of *MIPS* to develop low phytate plants. However, down-regulation of *MIPS* level results in pleiotropic effect like reduced apical dominance, enhanced leaf senescence, reduced yield^18^, reduced levels of ascorbate, inhibition of seed germination along with higher sensitivity to ABA in transgenics^3,19^. Therefore, a clarity on distinct role of *MIPS* during growth and immunity remains unclear.

To investigate the role of *MIPS* during growth and immunity, we analysed the *TaMIPS* gene family and characterized *TaMIPS-B* over-expressing rice transgenics. Our investigation suggests involvement of MIPS in ethylene synthesis and signaling response. An increase in expression of *MIPS* evokes ethylene response. Thus, we conclude that differential *MIPS* levels are required for normal growth, immunity and abiotic stress tolerance.

## Results

### Evolutionary conservation of *MIPS*

**Figure 1:**
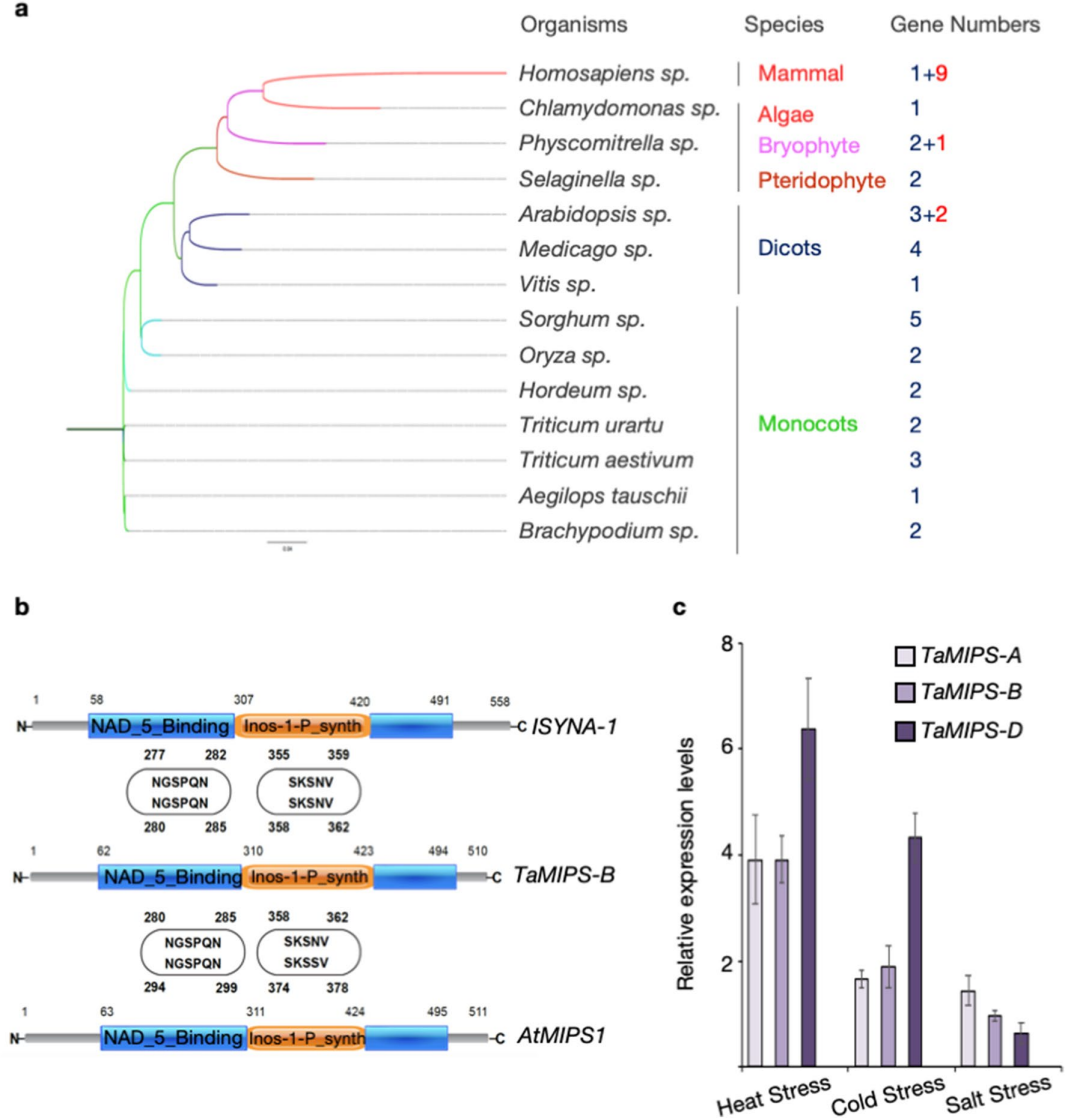
Gene Family and Expression Analysis of MIPS. (**a**) Phylogenetic analysis of major *MIPS using *ClustalOmega. Phylogenetic tree was build by Neighbour-joining method. Number of genes present in the gene family are represented by red coloured digit and isoforms in green coloured digits. (**b**) Schematic representation of conserved domains and motifs present in the *MIPS. *NAD_binding_5 superfamily and inositol phosphate synthase are colour coded by blue and orange regions, respectively . *TaM/PS-B *domains were compared to Human and *Arabidopsis *MIPS protein. (**c**) Relative expression levels of *TaMIPS-A, TaMIPS-B *and *TaMIPS-D *in 10-day-old seedlings during various simulated abiotic stress treatment.

To determine the evolutionary conservation in diverse species, we identified *MIPS* gene family in chosen organisms which covers a wide range of classes like mammals, bryophytes, pteridophytes, algae, monocots and dicots by HMM search. We fished out same number of MIPS proteins sequences in *Arabidopsis* and *Oryza*^20^. Out of all the members from the gene family of respective organisms, primary enzyme coding sequence was used for multiple sequence alignment by ClustalOmega and phylogenetic tree was created using Neighbor-joining method (Fig. 1a). We found an extensive sequence conservation with presence of identical amino-acids in *MIPS* genes with two most conserved motifs i.e. (NGSPQN) and (SKSNV) in all the organism (Fig. 1b) (Fig. S1). We also performed a domain search in these sequences, identified the NAD_5_Binding (PF07994) and Inos-1-P_synth domain (PF01658). Overall this investigation suggests the significant evolutionary conservation of (NGSPQN) and (SKSNV) motifs in the NAD_5_binding and Inos-1-P_synth domain, respectively.

### *TaMIPS* gene family, organization and expression analysis

We identified and isolated three MIPS transcript in wheat. All three *TaMIPS* gene are on wheat chromosome 4 of the A, B and D genome, so they were named *TaMIPS-A*, *TaMIPS-B*, *TaMIPS-D* according to their location. It was interesting to observe that *MIPS* gene family in *Triticum aestivum* consist of three genes only. Gene structure of MIPS homologues consist of 10, 9 and 10 exons in *TaMIPS-A*, *TaMIPS-B* and *TaMIPS-D*, respectively (Fig. S2). We further analysed the 2 Kb upstream sequence *TaMIPS-B* using PLACE database and found the regulatory elements related to light, SA and ET/JA responsive (Table S2). We also checked the homology between the *TaMIPS* transcripts and observed more than 99% homology between them. To investigate the differential expression of *TaMIPS*s homologs under various abiotic stresses, we carried out the quantitative RT-PCR in different stresses. We found elevated expression of *TaMIPSs* during heat stress which was prominent in *TaMIPS-D* (sixfold change) whereas *TaMIPS-B* and *TaMIPS-D* showed same expression compared to control condition (Fig. 1c). During cold stress, *TaMIPS-D* was found to have highest expression (fourfold change) among other members and followed by *TaMIPS-B* compared to control condition (Fig. 1c). We also investigated the expression of *TaMIPS* during salt stress and found that *TaMIPS-A* had highest expression amongst the three homologues followed by *TaMIPS-B* and *TaMIPS-D*. From this analysis, it appears that *TaMIPS-A, TaMIPS-B* and *TaMIPS-D* are stress inducible and *TaMIPS-B* was further characterized.

### *TaMIPS-B* overexpression imparts thermotolerance in rice

**Figure 2:**
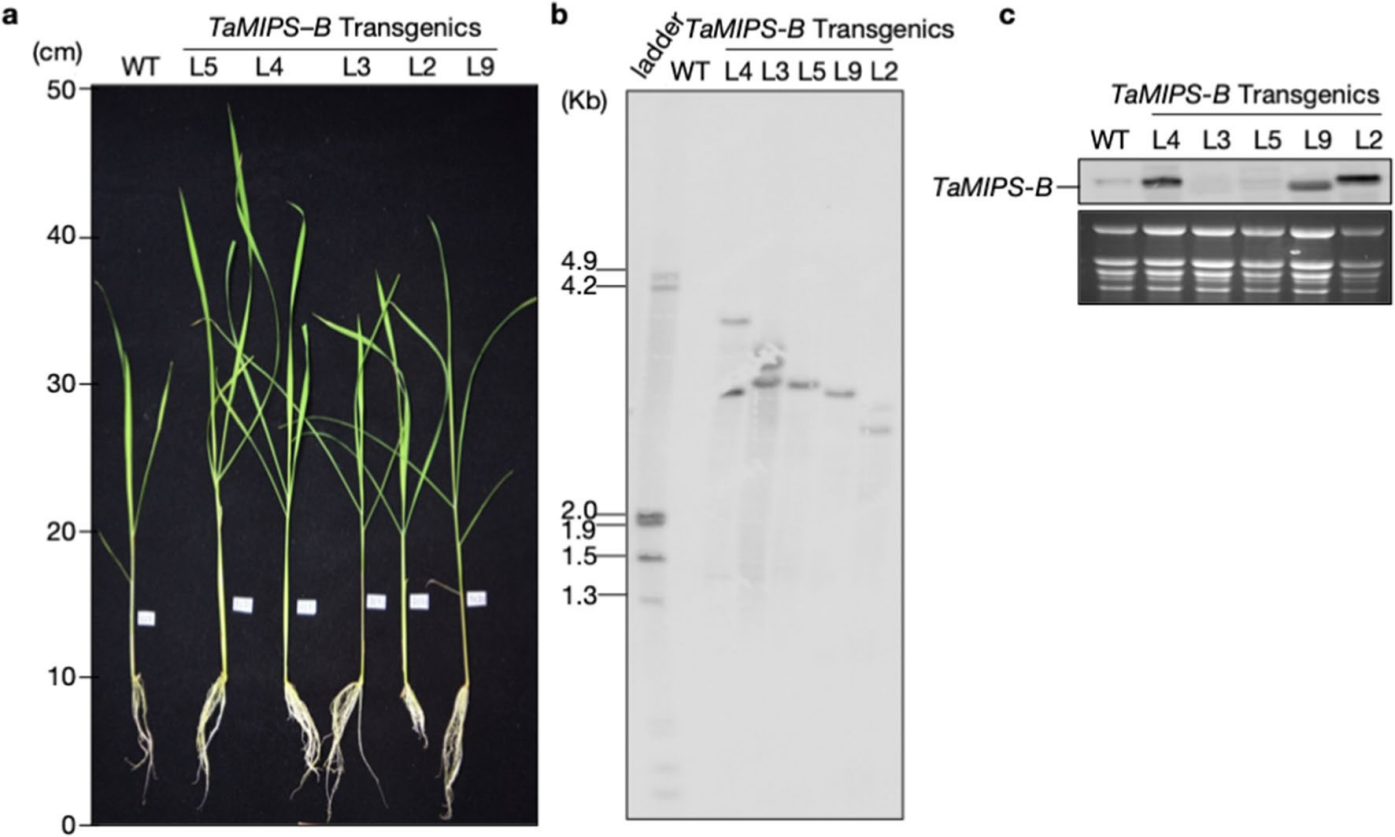
Phenotypic and molecular characterization of *TaMIPS-B *overexpression (OE) Rice T2 Transgenics. (**a**) Phenotype of 40-day-old *TaMIPS-B *overexpression rice T2 transgenics. (**b**) Southern blot analysis for gene copy number in *TaMIPS-B *overexpression rice T2 transgenics using *hpt *probe. (**c**) Northern blot analysis of *TaMIPS-B *overexpression transgenics plants using *TaMIPS-B *gene probe.

To characterize the *TaMIPS-B*, we raised the *TaMIPS-B* over-expressing rice transgenics. Of the various regenerants; at least independent 46 transgenic plants were identified, and five lines were chosen for detailed characterization (Fig. 2a). Southern blot analysis was carried out which revealed that transgenic line L5, L9 and L2 had a single copy insertion, while transgenic line L4 and L3 possess 2 and 3 transgene copies, respectively (Fig. 2b). Subsequent, expression analysis of *TaMIPS-B* transgenic lines was carried out and expression was observed in L4, L9, L2 whereas no signal was observed in L3 and L5 (Fig. 2c). This study suggests the expression of transgene in transgenic line L4, L9, L2 and no expression in L3 and L5. Transgene expressing Lines (L4, L9, L2) and silenced line (L3, L5) were used for further analysis and thermotolerance assay.

**Figure 3:**
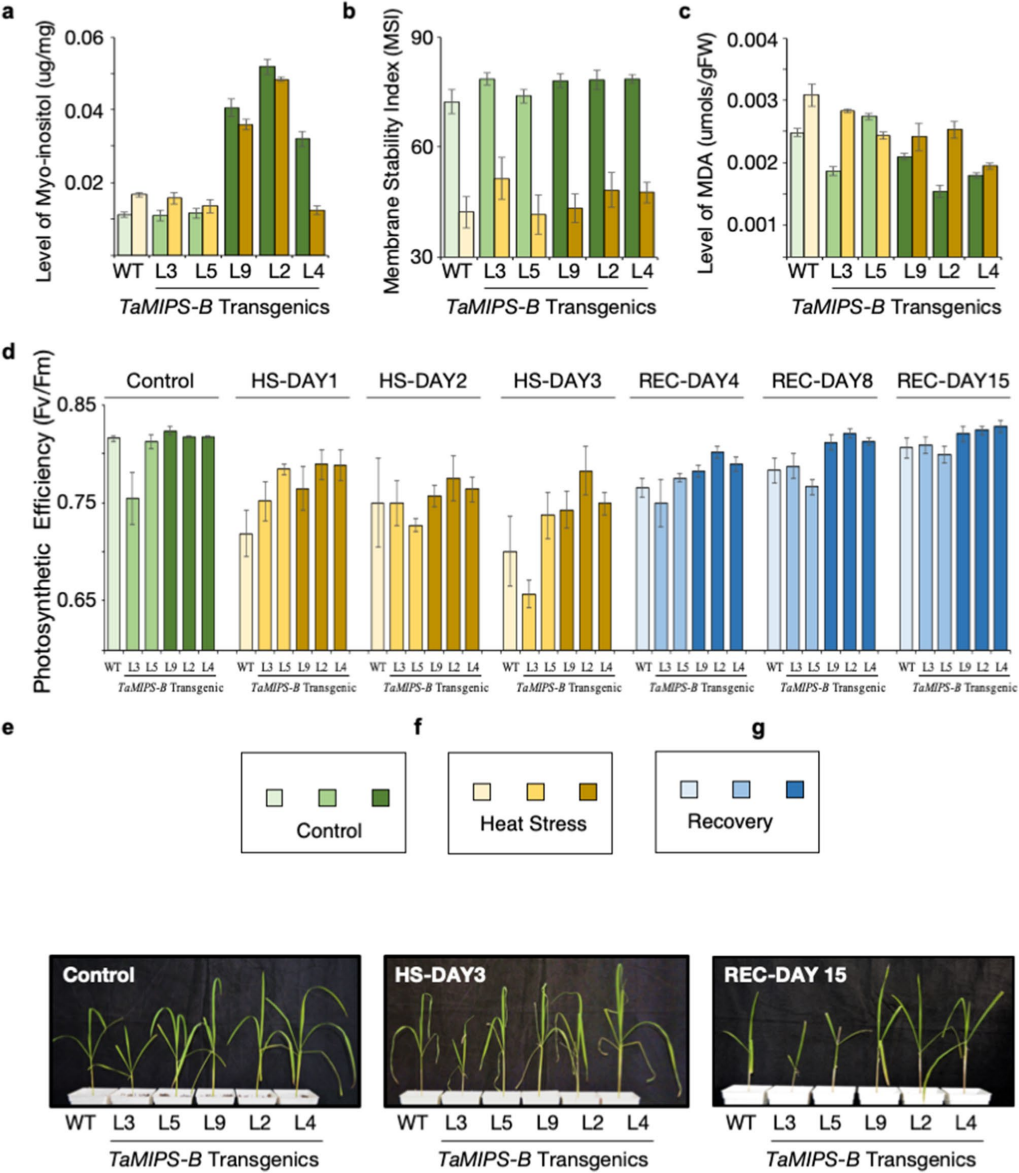
Physiological and Phenotypic Analysis in *TaM/PS-B *OE Rice T2 Transgenics During Heat Stress (HS). (**a**) Quantitation of Myo-inositol, (**b**) Membrane stability index (MSI), (**c**) MDA levels in wild type and *TaMIPS-B *transgenics under control and heat stress conditions, respectively. Error bar represent the ± standard deviation of five biological samples. (**d**) Photosynthesis efficiency (F_v_/F_m_) analysis of *TaMIPS-B *rice transgenics. Graph panel with green bar represent the photosynthetic efficiency of overexpression *TaM/PS-B *transgenics and wild type under control condition, yellow bar during heat stress and blue bars during recovery. Error bar represent the ± standard deviation of five biological samples. (**e–g**) Phenotypic analysis of *TaMIPS-B *overexpression transgenic during heat stress. (**e**) Photograph of representative plants of each line (L3, L5, L9, L2, L4) under control conditions, (**f**) After 3 days of heat stress at 45°C ±1 for 6 h, and (**g**) After 5 days of recovery in green house.

40-days old seeding were subjected to 45 °C ± 1 for 6 h consecutively for three days to assess the possible role of *TaMIPS-B* in heat stress. Control and treated plant samples of wild type and transgenics were collected to carry out the *myo*-inositol, MSI and MDA quantification. As expected, we found a significant increase in *myo*-inositol levels in the transgenic lines L9, L2, L4 as compared to the wild type with maximum levels in line no 2. Further, upon heat stress a similar profile was observed however, a decrease in levels of *myo*-inositol was seen in lines L9, L2, L4 during heat stress whereas an increase in line L3, L5 (Fig. 3a). Membrane stability index (MSI) was also measured and MSI was high in transgenic lines L3 (78%), L9 (77%), L2 (77%), L4 (78%) as compared to wild type (72%) under control condition (Fig. 3b). During heat stress, transgenics line L3 and L2 showed highest membrane stability followed by L4, L5. Wild type plants had least membrane stability upon heat stress. To determine the effect of heat stress, we checked the lipid peroxidation by quantifying the levels of MDA and found higher level of MDA in wild type plants upon heat stress as compared to the transgenic lines (Fig. 3c).

Photosynthetic efficiency of transgenics was also investigated under control and heat stress conditions. For heat stress treatment, 40-days old plants were subjected to 3-days heat treatment at 45 °C ± 1 for 6 h and transferred to green house after every treatment. Under control condition, F_V_/F_M_ values were higher in transgenics as compared to wild type plants (0.816) with maximum value in transgenic line L9 (0.823) followed by L2 (0.817), L4 (0.817) and L5 (0.812) whereas L3 showed least value (0.75) (Fig. 3d,e). During heat stress regime, F_V_/F_M_ values were measured and a sharp decrease in wild type plants was observed whereas transgenic lines L9, L2, L4 showed consistent tolerance as correlated by high value of F_V_/F_M_. L3 showed decrease at day three with no change till second day of heat treatment whereas L5 showed continuous decrease after first day of heat treatment (Fig. 3d,f). After heat treatment, plants were shifted to green house and observed that transgenics recovered much faster than the wild type as F_V_/F_M_ value of L2 reverted to 0.80 after three days followed by L4 (0.79) and L9 (0.78) (Fig. 3d,g). After 15 days of recovery, transgenic lines L9, L2, L4 recovered to their initial F_V_/F_M_ ratio but transgenic lines L2 and L4 after recovery showed increase in F_V_/F_M_ ratio compared to their control condition values.

### Reduced grain yield upon overexpression of *TaMIPS-B* in rice

**Figure 4:**
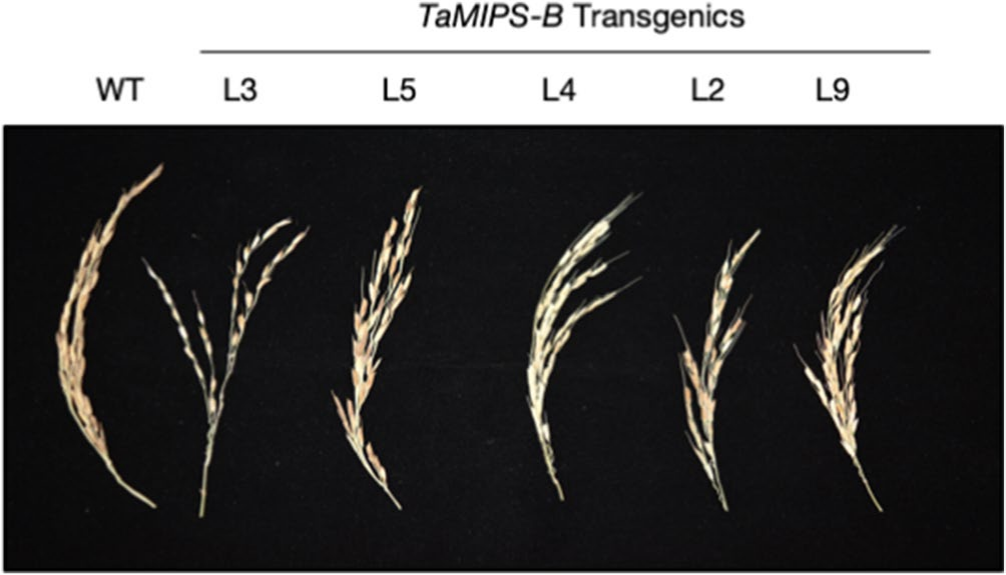
Phenotype of panicles *TaMIPS-B *overexpression rice T2 transgenics.

When the effect of *TaMIPS-B* overexpression on reproduction was analysed. The seed setting rate in panicle of *TaMIPS-B* transgenics and wild type (Fig. 4) was calculated. We observed a decrease in seed setting rate in all transgenic lines i.e. L9, L3, L4, L2 and a lowest percentage in L5 as compared to the wild type plant (Table 1). Reduced seed-setting rate suggest the negative effect of *MIPS* overexpression.

**Table 1:** Seed-setting rate of *TaMIPS-B* overexpression rice T2 transgenics compared with wild type.

**Plant genotype**	**Total floret **	**Fertile florets **	**Sterile florets**	**Seed setting rate (%)**	**% Decrease**
WT	431	237	194	54.9	NA
L3	278	105	173	37.7	31
L5	424	75	349	17.6	67
L9	505	159	346	45.4	17
L2	382	106	276	27.7	49
L4	255	116	139	31.4	42

### Gene expression analysis revealed enrichment defense pathway in *TaMIPS-B* transgenic

**Figure 5:**
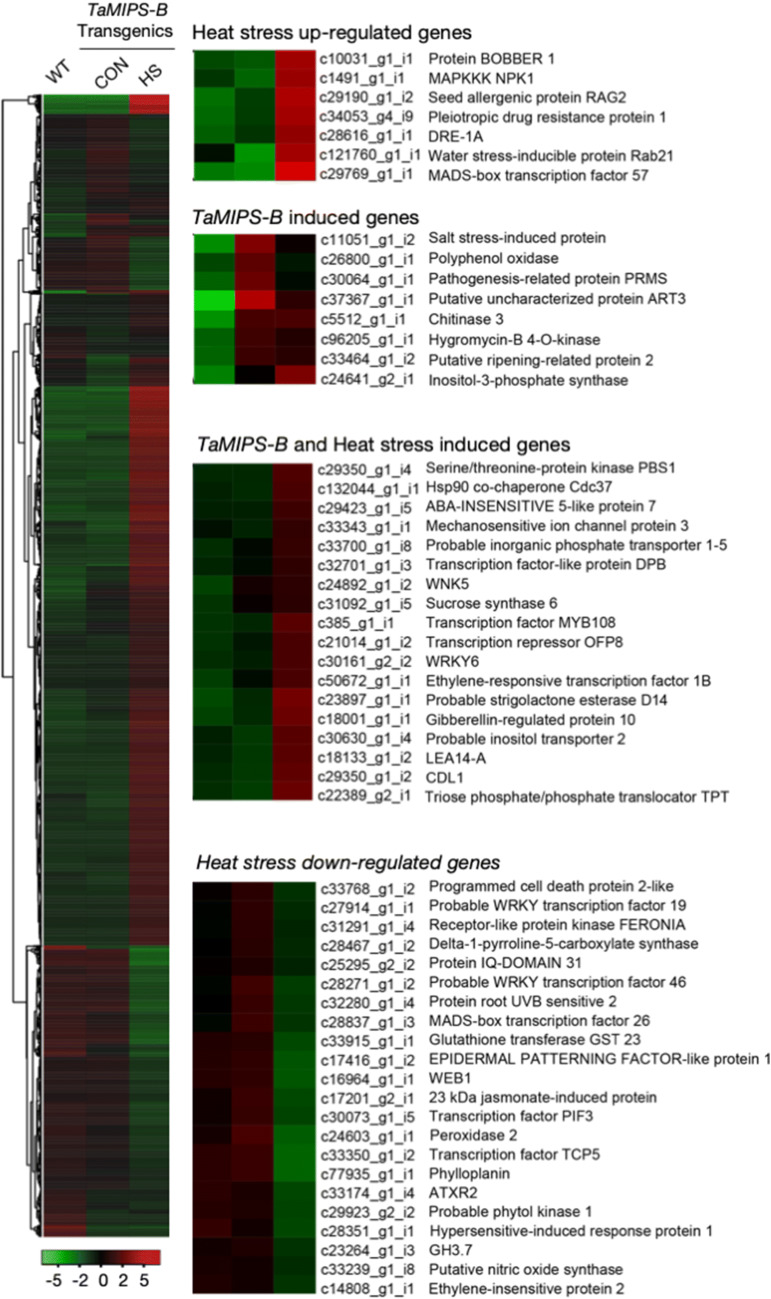
Expression analysis of *TaMIPS *and HS induced genes in rice transgenics. Heat map displaying differentially expressed genes of overexpression *TaMIPS-B *rice T2 transgenic based on log ratio of FPKM data. Column corresponds to the experimental samples and row corresponds to gene. The color key represents FPKM normalized log10 transformed counts. Red color corresponds to high expression and green to low expression. Representative TaM/PS-mediated up and down-regulated genes are highlighted.

**Figure 6:**
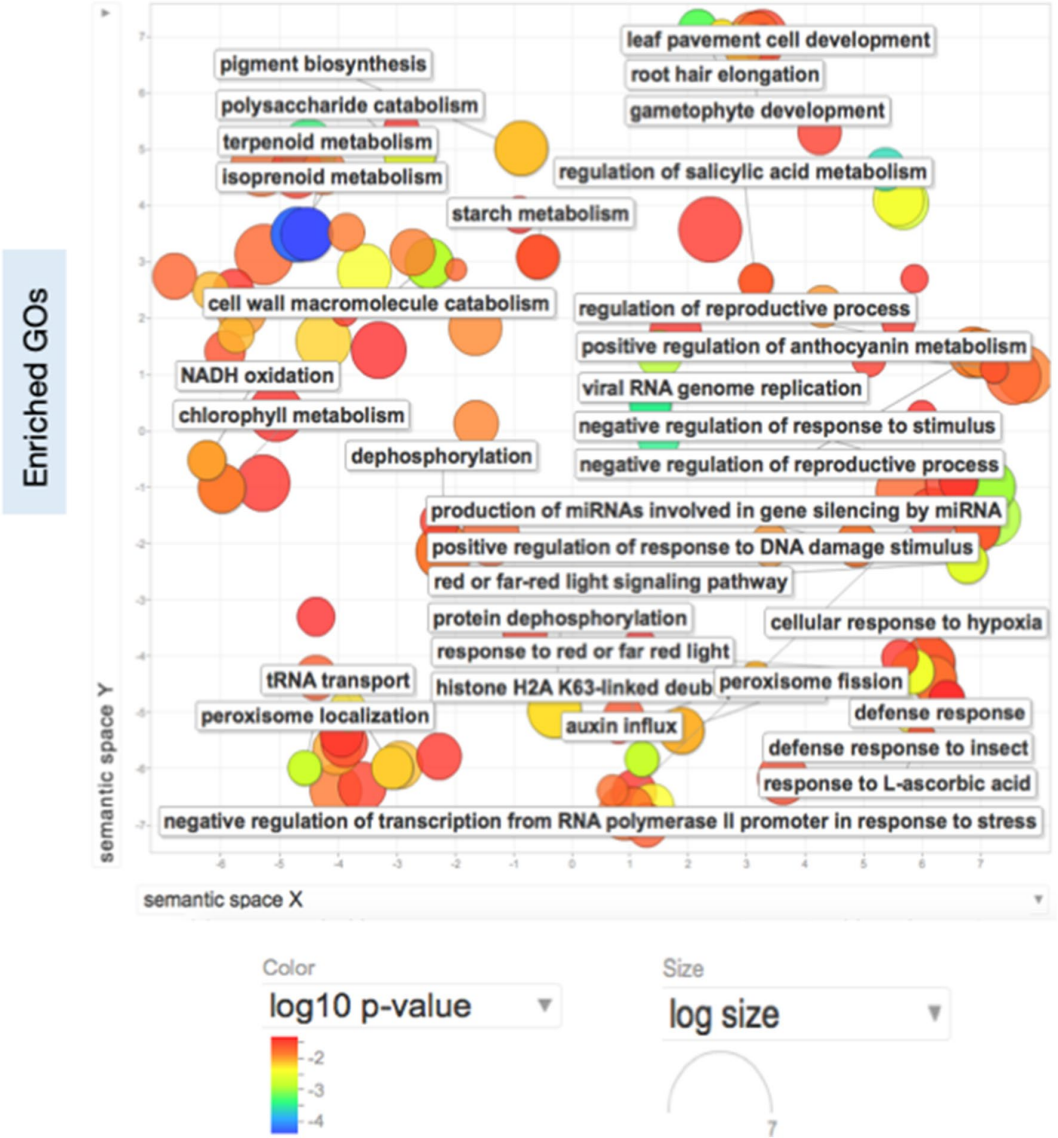
REVIGO scatterplot for visualizing enriched GOs. Enriched GOs in *TaMIPS-B *transgenics. REVIGO (reduce and visualize GO) scatterplot comprehends the overrepresented GO biological process categories in *Atmips1 *and *TaMIPS-B. *GO enrichment analysis was performed by Trinotate at P value s 0.05. Bubble colour corresponds the p-value obtained from GO enrichment analysis, whereas the bubble size corresponds to the GO term prevalence in the UniProt-GOA database.

To understand the role of MIPS in reduced reproductive potential, we carried out the whole transcriptome analysis of *TaMIPS-B* over-expressing transgenic under control and heat stress condition using Illumina HiSeq 2,500 sequencer with PE100 which resulted in generation of 57 to 70 million reads per sample. Paired end reads were assembled into 178,134 transcripts and 66,106 genes in *TaMIPS-B* over-expressing transgenics with N50 of 2,249 by Trinity. Finally, a total of 5,879 transcripts were found to be differentially expressed in the *TaMIPS-B* transgenics. Interestingly, we observed that the up regulation of defense genes like chitinase3, WRKY transcription factor 6, Pathogenesis-related protein PRMS along with ERF1B in *TaMIPS-B* over-expressing transgenics (Fig. 5). We carried out the gene ontology (GO) term enrichment analysis using upregulating genes in the *TaMIPS-B* over-expressing rice transgenic. To reduce the GO terms, we used REduced VIsualize Gene Ontology (REVIGO)^21^. We found multiple GO term like “defense response”, “defense response to insect” and “regulation of salicylic acid metabolic process” (Fig. 6).

### Identification of MIPS interacting protein in wheat

**Figure 7:**
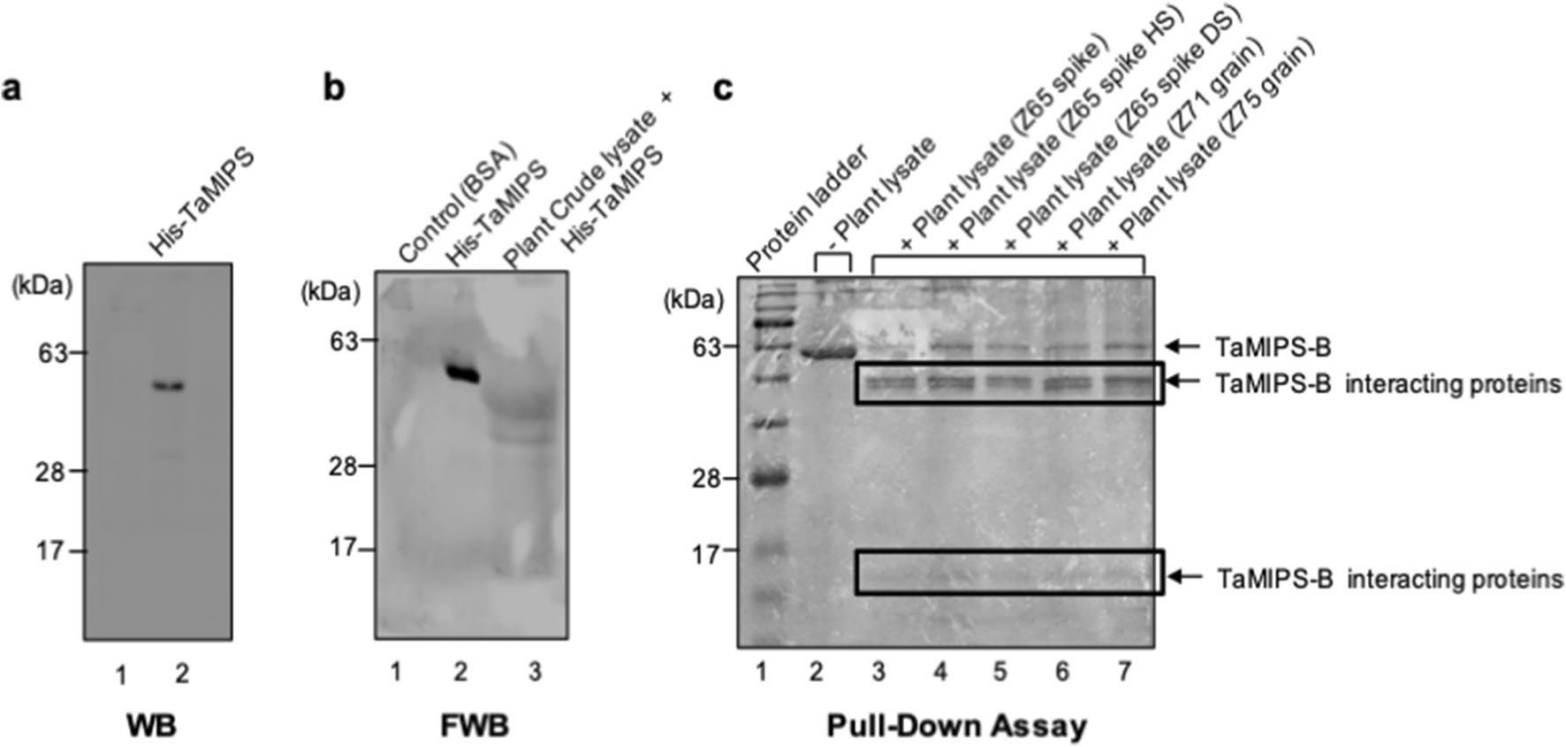
Identification of TaMIPS-B lnteractors. (**a**) Western blot analysis of His-TaMIPS-8 recombinant protein with anti-His antibody. (**b**) Far-western blot analysis was done using 50ug of purified recombinant protein. After extensive washes, interacting protein were detected using anti-His antibody and visualized with lmmobilonTM western (Millipore). (**c**) Isolation of TaMIPS-8 interacting proteins from plant lysates by in-vitro pull-down assay. SDS_PAGE (12%) demonstrating, a putative interactors of TaMIPS-8 protein.

To gain insight into the mode of action of MIPS, we investigated the presence of interacting proteins of TaMIPS-B as previous reports suggest its dual nature. We therefore performed the far-western analysis using purified His-TaMIPS-B protein (Fig. 7a) and crude plant extract and found two interacting signals in the range of 35–48 kD (Fig. 7b). Further, we performed in-vitro pull down using recombinant His-tagged TaMIPS-B expressed in bacteria *E. coli*. The protein obtained were separated by SDS page and Coomassie stained. We found three more protein bands along with the recombinant His-TaMIPS-B in all the plant samples except four bands in grain_Z75 plant sample. Z75 plant sample was used for further analysis. Out of four bands, three were in size range of 48 kDa to 35 kDa (Fig. 7c).

We observed the presence of MIPS sequence in the first two bands which range in 48 kDa to 35 kDa (Table 2). This result suggests TaMIPS-B interacts with its homologs as molecular weight of TaMIPS-B and TaMIPS-D were in this range (Table S1). Further pull-down extract was subjected to LC–MS analysis to identity the interacting proteins. We found ethylene related proteins (Table 3).

**Table 2:** Identification of MIPS proteins by MALDI-MS.

**Band number**	**Accession Id**	**Protein name**	**Peptide sequence**
1	GI: 14548089, GI: 1040821086	MIPS	VQQANYFGSLTQASTIR, DKVQQANYFGSLTQASTIR, VQQANYFGSLTKASTIR
2	GI: 14548089	MIPS	VQQANYFGSLTQASTIR, DKVQQANYFGSLTQASTIR
3	No	No MIPS hit	NA
4	No protein identified	No protein identified	NA

**Table 3:** List of probable interactors of TaMIPS-B identified by MS/MS.

**Swiss-Prot accession number**	**Protein name**	**Theoretical mW (Da)**	**PLGS score**	**Sequence coverage (%)**
G8HMZO	Myo-inositol-1-phosphate synthase	56086	828	21.7
M7YZ59	1-aminocyclopropane-1-carboxylate oxidase 3	33023	201.3	11
M7ZRU2	Serine/threonine-protein kinase CTR1	133646	34.3	5
W5AA37	Peroxidase	36713	26.5	2.93

## Discussion

Myo-inositol has been associated with numerous roles i.e. cell wall biogenesis^1^, auxin storage^2^, phytic acid synthesis^3^, and oligosaccharides synthesis^4^. In this study, we have proposed that *MIPS* acts as a molecular switch to control growth and immunity.

*MIPS* gene family analysis in organism included in this study indicates their less diversification due to the presence of a relatively small gene family which usually consist of 1–5 genes. We identified previously reported conserved motifs (NGSPQN) and (SKSNV) in all the major *MIPS* coding gene from all the organism studied. MIPS is a phosphoprotein and its activity is regulated by phosphorylation at serine residue. Phosphorylation of serine residue of (NGSPQN) is predicted via GSK-3 which increases the MIPS activity whereas phosphorylation of serine residue of (SKSNV) via PKC decreases the activity^6^. Conservation of above said motifs across species including *Homo sapiens*, suggests a common mechanism of MIPS enzyme regulation at post-translational level.

Expression analysis of *TaMIPS* in whole seedlings suggests differential expression of *TaMIPS-B*, *TaMIPS-A, TaMIPS-D* in wheat during induced stress conditions. *TaMIPS*s expression during stress revealed the stress inducible behavior of *TaMIPS-A*, *TaMIPS-B* and *TaMIPS-D* with highest expression of *TaMIPS-D* in thermal stress (heat and cold stress) whereas *TaMIPS-A* showed highest expression under salt stress. Similarly, high expression of *AtMIPS2* in heat and *AtMIPS3* in heat and drought has been seen in previous report where the *AtMIPS1* showed less expression compared to *AtMIPS2* and *AtMIPS3*^20^. Differential expression of *CaMIPS1* and *CaMIPS2* also demonstrated the similar situation in which *CaMIPS2* was induced by environmental stress with no effect on *CaMIPS1*^22^.

We next carried out generation and physiological analysis of *TaMIPS-B* over-expressing rice transgenics. We observed two and three copy number of transgenes in L4 and L3, respectively. However, transgene expression was observed in transgenic lines L9, L2, L4 but no expression in L3 and L5. The probable reason could be the high copy number in case of L3 which led to the silencing whereas in L5 DNA methylation, position effect, trans-inactivation could be the reason of gene silencing. Previous report correlates high transgene copy number with transgene silencing in tobacco^23^. Moreover, a recent study also demonstrated the overexpression of *AtMIPS2* resulting in co-suppression of all the three *AtMIPS* genes which leads to reduced root length, delayed or absence of flowering along with reduced fertility^24^. Further these transgenics were used for thermotolerance assay. As expected, we observed increase in *myo*-inositol in transgenic line L9, L2, L4 with maximum level in L2 in control condition. A similar pattern was observed during heat stress but with slight decrease as compared to control conditions. When MDA (malondialdehyde) levels were checked in transgenic and wild type, we found low MDA levels in all transgenic line except L5. During heat stress, MDA levels increased in all the lines but the level of MDA in transgenics was lower as compared to wild type. MSI too has been correlated with heat tolerance under heat stress regime in wheat^25^. We observed higher MSI in transgenic line L9, L2, L4 along with L3 during heat stress and control condition compared to wild type.

Heat stress also affects respiration and photosynthesis which results in decrease in plant productivity^26^. During heat stress regime, transgenic lines performed better especially transgenic line L2 which had the highest *myo*-inositol level at heat treatment day 3. High level of *myo*-inositol could be correlated to high photosynthetic efficiency of PSII. During recovery period after heat stress, transgenic lines recovered completely after 7 days whereas wildtype along with transgene silenced line L3 and L5 could not recover to control F_v_/F_m_ ratio. Phenotypic analysis also showed a similar pattern; transgene silenced lines and wild type could not recover from the heat stress, even after 15 days of recovery whereas transgene expressing lines L9, L2, L4 showed growth and emergence of new leaves as early as 7 days of recovery in L2. Over-expression of *SaINO1* showed significantly enhanced tolerance during salt stress in *Arabidopsis*^9^, whereas Ectopic expression of *RINO1* in rice and overexpression of *IbMIPS* in sweet potato results in enhanced tolerance to salt and drought stress^10,15^. Previous report from our lab also demonstrated that overexpression of *TaMIPS-B* in *Arabidopsis* confers heat tolerance^27^ and the present data also supplements our assumption that over-expression of *TaMIPS-B* results in enhanced heat tolerance in this instance in rice.

AtMIPS1 has been reported as metabolic enzyme and a transcriptional regulator^28^. Previous study has also revealed the existence of human MIPS as an enzyme complex with its isoforms and these complexes are tailored according to the tissue specific needs^8^. Our Far western and pull-down data suggests TaMIPS-B interaction with its homologues as well as 1-aminocyclopropane-1-carboxylate oxidase 3, serine/threonine-protein kinase CTR1 and might affect their activity as well. Therefore, we conclude that TaMIPS-B interacts with these proteins and forms an enzyme complex to carry out its function in the cell. Moreover, our results are substantiated by *Arabidopsis* interactome study which has predicted the interaction of AtMIPS1 with AtMIPS2 along with peroxidase and AtMIPS2 with ACO3^29^.

We also performed whole transcriptome analysis of *TaMIPS-B* transgenics and further gene ontology analysis which revealed activation of defense response. We observed the up-regulation of ERB1B in transgenics. ERF1B is considered as an integrating point for ethylene and jasmonate signals and activates the defense related gene like chitinase 3, 23 KDa jasmonate-induced protein, ß-glucosidase, peroxidase 2, WRKY transcription factor 6, glutathione S-transferase T3 and thaumatin-like protein which were reported to be upregulated in overexpression Col-0;35S:ERF1 transgenics^30^. Up-regulation of these genes in *TaMIPS-B* transgenic suggests the activation of ET/JA signaling. Overexpression of *IbMIPS1* provides resistance to stem nematode^15^, presence of elements related in ET/JA in *MIPS* promoter and GO term “defense response to insect” supports the activation of ET/JA pathway. From this data, we can conclude that the up-regulation of *MIPS* results in activation of ET/JA–dependent defense pathway in *TAMIPS* over-expressing rice transgenics.

When we investigated *TaMIPS-B* over-expressing rice transgenic at reproductive stage and we observed a reduced in seed setting rate with maximum reduction in L2 which had highest level of MI. During heat stress, rate of evolution of ethylene in heat sensitive variety is higher than tolerant one, which is correlated to higher male sterility and results in reduced grain yield^31,32^. Decrease in seed setting rate in over-expressing *TaMIPS-B* transgenic might be because of over-production of ethylene which results in reduced fertility.

**Figure 8:**
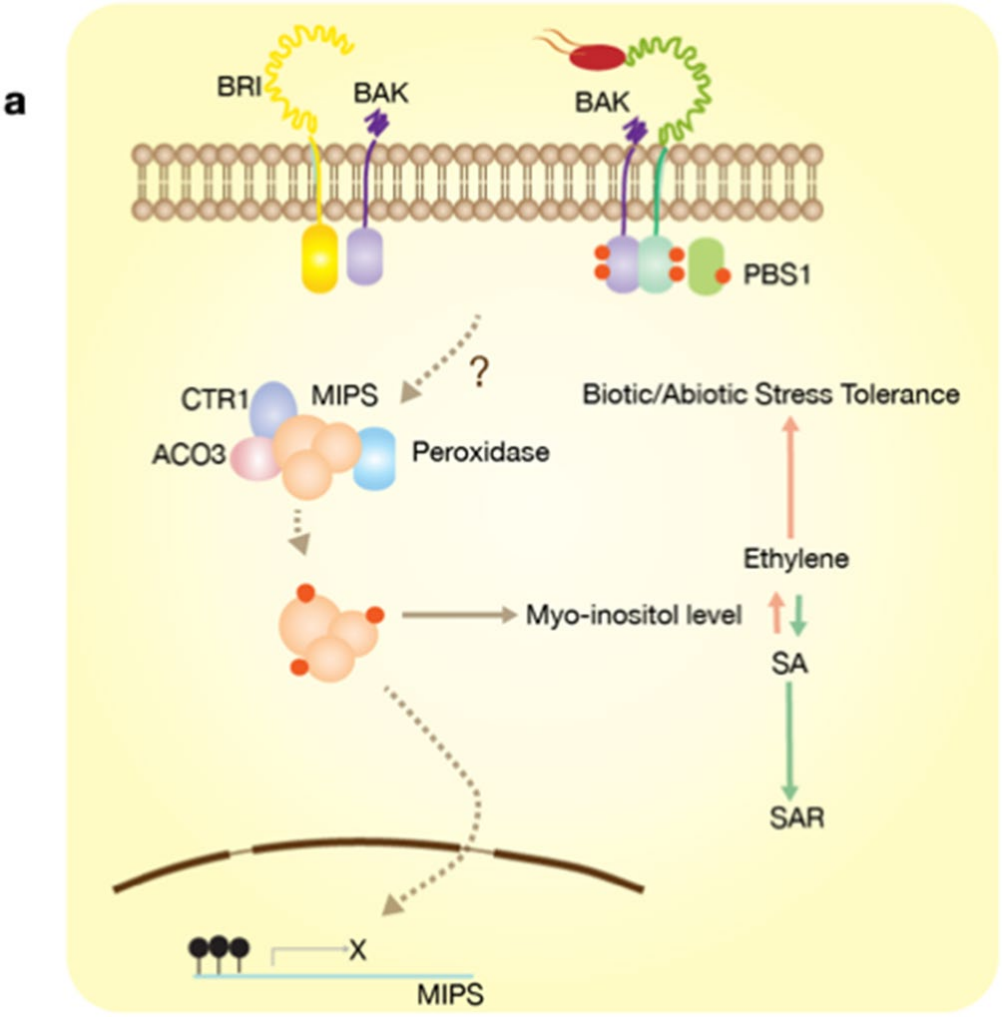
A model based on findings and published data. myo-inositol phosphate synthase (MIPS) in regulating the stress response. During normalconditions, optimal level of Ml are maintained and when the host encounters abiotic or biotic stress conditions, it increases or decreases Ml level to activate ethylene response or SA­mediated defense responses depending upon nature of stress, type of stress and causative agents.

In conclusion, activation of SA defense response in *Atmips1* and overexpression of *IbMIPS1* provides resistance to stem nematode and salt stress^15,17^ suggests us that plants perceive altered levels of myo-inositol as a stress effector molecule and result in activation of defense response. We proposed the hypothesis that receptors like kinases might be phosphorylating the serine residue of (SKSNV) motif which result in rapid increase or decrease in activity of myo-inositol phosphate synthase^6^ and subsequent increase and decrease in level of MI is perceived as the stress condition. We can thus conclude that during normal conditions optimal level of MI are maintained and when the host encounters abiotic or biotic stress conditions, it increases or decreases MI level to activate ethylene response or SA-mediated defense responses depending upon the stress, type of stress and causative agents. It is therefore one of the important central regulators in balancing plant growth and immunity (Fig. 8).

## Materials and methods

### *TaMIPS* gene mining

Protein and nucleotide sequences were downloaded from ftp://ftp.ensemblgenomes.org/pub/plants/release22/fasta/triticum_aestivum/, https://www.ensembl.org/info/data/ftp/index.html and https://plants.ensembl.org/info/website/ftp/index.html. *TaMIPS* genes were identified in protein sequences by HMM search (https://hmmer.org) using inositol phosphate synthase (Inos-1-P_synth) domain hmm from Pfam database with default E-value of 10. Similarly, MIPS sequences from other organisms i.e. *Aegilops tauschii, Arabidopsis thaliana, Brachypodium distachyon, Chlamydomonas reinhardtii, Hordeum vulgare, Medicago truncatula, Oryza sativa, Physcomitrella patens, Selaginella moellendorffii, Sorghum bicolor, Triticum urartu, Vitis vinifera* and human were identified.

### In-silico analysis of *TaMIPS* gene family

*TaMIPS* gene structure was predicted by Splign (https://www.ncbi.nlm.nih.gov/sutils/splign). Protein Sequences of *TaMIPS* were checked for the domains using NCBI Conserved Domain Database (NBCI-CDD)^33^ and motif search tool (https://www.genome.jp/tools/motif/). To deduce the phylogenetic relationship between the homologous *TaMIPS*, protein sequences were aligned by ClustalOmega (https://www.ebi.ac.uk/Tools/msa/clustalo/) and phylogenetic tree was made using Neighbour-joining method. Phylogenetic tree was drawn using FigTree program.

### Plant sample, growth and stress conditions

Wheat (*Triticum aestivum*) seeds (PBW 343) were surface sterilized by sodium hypochlorite for 20 min and grown at 22 ± 1 with 16:8 light and dark cycle for 10 days. 10-day-old seedlings were used for expression analysis under different stress conditions. For temperature stress, seedlings were exposed to 42 °C and 4 °C for 4 and 24 h respectively. For salt stress, seedlings were transferred to 200 mM NaCl solution for 24 h.

Rice transgenics were raised using *Oryza sativa* ssp. Indica var. PB1. Sterilized seeds were used for callusing and transgenic generation was done according to protocol described by Nishimura et al.^34^. Regenerated plantlets were then transferred to a rooting media and subsequently to pots and raised to maturity.

### RNA isolation and cDNA synthesis

RNA was isolated from the different plant tissues (*TaMIPS-B* over-expressing rice transgenic seedling, control and stressed sample) using RNeasy plant mini kit (Qiagen, Germany). Samples were ground with liquid nitrogen and further proceeded according to the kit manual. In-column DNase treatment was done to remove the genomic DNA contamination. Quality and quantity of RNA samples were done by gel electrophoresis and nanodrop. 2 μg of RNA was used to make the cDNA using High-Capacity cDNA Reverse Transcription Kit (ThermoFisher Scientific) and SuperScrip III First-Strand Synthesis System for Full length cDNA synthesis.

### Cloning of *TaMIPS-B* CDS

*TaMIPS-B* full length cDNA was amplified from cDNA (forward primer AAGGATCCATGTTCATCGAGAGCTT, reverse primer AAGGTACCTCACTTGTACTCCAGGAT) was cloned in pB4NU vector via restriction digestion by BamH1 and Kpn1*.* Clone was confirmed by restriction digestion and DNA sequencing and the confirmed clone was used to transform rice calli using EHA 105 strain of *Agrobacterium tumefaciens* harboring pB4NU-*TaMIPS-B*.

### Promoter sequence analysis

Promoter sequence of *TaMIPS-B* was fetched from IWGSC chromosome assemblies using CLC genomic work bench. A 2 kb putative promoter sequence was searched against PLACE *cis* elements database^35^ using CLC genomic workbench.

### Genomic DNA isolation and southern analysis

100 mg fresh leaves transgenics were homogenized in liquid nitrogen and mixed with 500 μl CTAB buffer (2% w/v CTAB, 0.3 M NaCl, 20 mM EDTA, 100 mM Tris–Cl (pH-8) 0.2% BME). Genomic DNA was isolated according to Murray and Thompson^36^. Quality and quantity of genomic DNA was checked by gel electrophoresis and nanodrop (GE, US), respectively.

Transgene copy number was checked using 10 μg of genomic DNA. DNA was digested with BamHI and separated by 0.8% agarose gel electrophoresis. DNA was blotted on Hybond-N Nylon membrane (Pharmacia) and fixed using UV cross linker (UVP HybriLinker, Analytik Jena, US). Blot was probed with *hpt* gene probe which was synthesized using PCR DIG probe synthesis kit (SIGMA-ALDRICH). Hybridization and detection were done using the non-radioactive method by DIG Luminescent Detection Kit as per the manufacturer guidelines (SIGMA-ALDRICH) with hybridization temperature 42 °C and washing 65 °C. Detection was carried out using CSPD solution and visualized by Fujifilm LAS4000 luminescence imager.

### Northern analysis

Transgene *TaMIPS-B* expression was checked by Northern blot analysis in rice transgenics. Total RNA from leaves of selected transgenic lines was isolated. 15 μg of RNA was denatured at 65 °C for 5 min, separated on 1% formaldehyde agarose gel^37^ and probed with *TaMIPS-B* gene probe. Blotting, hybridization and detection was done as described above.

### Total plant protein extraction

Total plant protein was isolated from five wheat samples (PBW343) named spike zadok 65, heat stressed spike zadok 65, drought stressed spike zadok65, grain zadok 71, and grain zadok 75. 100 mg of leaves were homogenized in liquid nitrogen and mixed with 1 ml extraction buffer 50 mM NaHPO_4_, 150 mM NaCl, 0.5% Triton-X-100, 10% glycerol, 0.5 mM PMSF and 25 mM imidazole (IDZ). Samples were vortexed thoroughly followed by centrifugation at 10,000 *g* at 4 °C for 5 min. Total protein was collected from Supernatant and estimation of protein was done by plotting standard curve of BSA.

### Membrane stability index

The Membrane Stability Index (MSI) was determined as explained by Singh and Khurana^38^. *TaMIPS-B* over-expressing rice transgenics leaf tissue (control and stressed) were placed in MQ water and initial conductivity (C1) was measured after incubating for 30 min at RT. Samples were then autoclaved for 20 min under 120 psi pressure and C2 was measured. MSI was calculated according to MSI = (1-(C1/C2)).

### Photosynthetic yield

Photosynthetic efficiency of *TaMIPS-B* over-expressing rice transgenics was measured using a PAM-210 (H.Waltz, Germany) under control, heat stress and recovery period. To measure the Fv/Fm, plants were dark adapted for 30 min and measuring beam was applied to leaf to measure F_0_ followed by application of saturating pulse using the F_v_/F_m_ function to measure the F_m_ (maximum fluorescence). F_v_/F_m_ value calculated by instrument (PAM-210) using the formula as following: F_v_/F_m_ where F_v_ = F_m_-F_o_.

### MDA quantification

Lipid peroxidation level in wild type and *TaMIPS-B* over-expressing rice transgenic plants was checked under control and stress conditions by quantifying the MDA content. 100 mg of tissue was homogenized by adding 500 μl of 0.1% (w/v) Trichloro acetic acid and centrifuged at max speed for 10 min and 500 μl of supernatant was mixed with 0.5% TBA (thiobarbituric acid) followed by incubated at 95 °C for 25 min. Reaction was stopped at incubating in ice and absorbance was measured at 532 and 600 nm.

### *Myo*-inositol quantification

myo-inositol levels were quantified using myo-inositol assay kit (Megazyme, USA). *TaMIPS-B* over-expressing rice transgenics leaf tissue were homogenized in MQ water and centrifuged at max speed for 5 min and reaction was set as per the kit instructions. Absorbance of samples were measured at 492 nm and myo-inositol content was calculated by the formula: Myo-inositol = 0.2046 × ∆A_Inositol_ where ∆A_Inositol=_ Absorbance 2 – Absorbance1.

### Transcriptome analysis

Whole transcriptome comparison of *TaMIPS-B* over-expressing rice transgenics was done against wild type. Paired-end cDNA library preparation was prepared from pooled RNA sample of three gene expressing chosen transgenic lines. RNA sequencing was carried out by SciGenom, India. De-novo assembly of Reads from each library was carried out using software package Trinity 2.0.6 with default parameters^39,40^. Generated transcripts were used for differential expression analysis. Transcript abundance estimation was done using the software RSEM 1.2.7 which outputs the expression-normalizing value FPKM. Differential expression was carried out using abundance matrix files generated by RSEM, by software EdgeR 2.14^41^ with the default parameters and TMM (Trimmed Mean of M-values) comparison was done across all the samples which resulted into a new matrix with normalized expression values across samples measured in FPKM. The expression patterns of the transcripts in the samples were restricted to the transcripts with significant differential expression (P-value 0.01, Fold change log2 scale).

### Functional annotation and gene ontology (GO) enrichment analysis

Assembled transcripts were annotated using Trinotate pipeline (https://trinotate.github.io/). Assembled transcripts were compared against Swissprot-Uniprot database, using blastn with an E-value 10^–5^. Transdecoder v.2.0.1 (https://transdecoder.sourceforge.net/) was used to predict the longest ORF in assembled transcripts. Predicted ORFs were then annotated using blastp against the Swissprot-uniprot database with an E-value cut-off of 10^−6^. Further annotation was carried out using Trinotate as per the manual. REVIGO scatter plot was used to visualization of GO enrichment analysis (https://revigo.irb.hr).

### Expression and purification of *TaMIPS-B*

*TaMIPS-B* gene was cloned into expression vector pET101/D-TOPO (ThermoFisherSCIENTIFIC). Cloning and transformation of cloned vector in BL21(DE3) was done as per the manufactural instructions (pET100/D-TOPO (ThermoFisherSCIENTIFIC). Large scale expression of recombinant protein was done at 16 °C by inducing the bacterial culture (0.5 OD) with 0.5 mM IPTG in BL21(DE3) cells. Bacterial pellet was lysed using lysis buffer (50 mM NaH_2_PO_4_, 300 mM NaCl, 1 mM PMSF, 0.1% Tween-20, 10% glycerol, 10 mM imidazole) and sonicated. Crude extract was cleared by centrifugation at 10,000 rpm at 4 °C for 30 min and used immediately. TaMIPS-B-His was purified using Ni–NTA beads with a 10-ml bed volume and 10 mg of crude TaMIPS-B-His extract. TaMIPS-B-His crude extract was incubated with Ni–NTA beads at 4 °C for binding for 1 h and washed extensively by washing buffer (50 mM NaH_2_PO_4_, 150 mM NaCl, 1 mM PMSF, 20 mM imidazole) and bound protein were eluted with an equal bed volume of elution buffer (50 mM NaH_2_PO_4_, 300 mM NaCl, 1 mM PMSF, 70 mM Imidazole, 10% glycerol) in 2 ml fractions. Cell lysate supernatant, insoluble pellet, eluted fractions were checked for recombinant protein by 12% SDS PAGE with Coomassie blue staining. Purified TaMIPS-His protein was then confirmed by western blot analysis using anti-His antibody and MALDI-TOF.

### Western and far-western assay

To check the TaMIPS-B protein interaction, Far-western analysis was carried out using purified TaMIPS-B-His recombinant protein as bait and plant crude extract (Zadok 65 spike stage) as prey according to Wu et al.^42^. 500 μg prey protein along with BSA (Bovine serum albumin) and purified His-*TaMIPS-B* as negative and positive control, respectively, were blotted on membrane in two sets and one of it was proceeded with western blot analysis and the other blot used for far-western analysis. Western blot was probed and detected by anti-His antibody whereas other was proceeded to denaturing/renaturing of blotted prey protein for 12–16 h. Blot was then blocked with 5% milk and washed with PBST (Phosphate buffered saline with Tween-20) buffer three times. Membrane was then incubated with bait protein (Purified His-TaMIPS-B protein) at 4 °C for 12 h. Bait protein bound to prey protein was detected on blot after stringent washing and incubating it with primary (anti-His) antibody followed by secondary antibody. Visualization was done by chemiluminescence using Immobilon Western Chemiluminescent HRP Substrate (MERCK) according to manufactural instructions using Fujifilm LAS4000 luminescence imager.

### Pull-down assays

Pull-down assay was preformed using the 500 μg of purified TaMIPS-B-His recombinant protein with 2 mg of crude wheat extract. Wheat extracts were prepared by homogenizing control samples of spike at Zadok 65 (spike_Z65) stage along with treatments i.e. heat and drought and grain sample (grain_Z71, grain_Z75) total plant protein extraction buffer (50 mM NaH_2_PO_4_, 50 mM NaCl, 1 mM PMSF, 0.1% Triton X-100). Homogenate was centrifuge at 5,000 rpm for 5 min and supernatant transferred to fresh MCT. Purified recombinant protein, Ni–NTA beads and plant crude extract were incubated at 4 °C with gentle shaking for 12 h. Beads were then centrifuged and washed extensively with washing buffer (same as above) and bound protein eluted with equal bed volume of elution buffer (same as above). The eluted protein was then subjected to SDS-PAGE electrophoresis and protein bands isolated and given for MALDI-TOF Mass Spectrometry (CIF, Biotech Centre, UDSC). Pull-down extract was also given for Liquid Chromatography Mass Spectrometry (LC–MS) analysis to Sandor, India.

### Statistical analyses

All values reported in this work are the average of at least two independent biological replicates having at least 15 seedlings. Error bars represent SE.

## Supplementary information

Supplementary file1 (PDF 315 kb)

## References

[1] Loewus FA, Kelly S, Neufeld EF (1962). Metabolism of *myo*-inositol in plants: conversion to pectin, hemicellulose, D-xylose, and sugar acids. Proc. Natl. Acad. Sci..

[2] Abreu EFM, Aragão FJL (2007). Isolation and characterization of a *myo*-inositol-1-phosphate synthase gene from yellow passion fruit (*Passiflora edulis* F. Flavicarpai) expressed during seed development and environmental stress. Ann. Bot..

[3] Ali N, Paul S, Gayen D, Sarkar SN, Datta SK, Datta K (2013). RNAi mediated down regulation of *myo*-inositol-3-phosphate synthase to generate low phytate rice. Rice.

[4] Karner U, Peterbauer T, Raboy V, Jones DA, Hedley CL, Richter A (2004). *Myo*-inositol and sucrose concentrations affect the accumulation of raffinose family oligosaccharides in seeds. J. Exp. Bot..

[5] Bachhawat N, Mande SC (2000). Complex evolution of the inositol-1-phosphate synthase gene among archaea and eubacteria. Trends Genet..

[6] Deranieh RM, He Q, Caruso JA, Greenberg ML (2013). Phosphorylation regulates *myo*-inositol-3-phosphate synthase a novel regulatory mechanism of inositol biosynthesis. J. Biol. Chem..

[7] Majumder AL, Johnson MD, Henry SA (1997). 1L-*myo*-inositol-1-phosphate synthase. Biochim. Biophys. Acta Lipids Lipid Metab..

[8] Seelan RS, Lakshmanan J, Casanova MF, Parthasarathy RN (2009). Identification of *myo*-inositol-3-phosphate synthase isoforms: Characterization, expression, and putative role of a 16-kDa γ_c_ isoform. J. Biol. Chem..

[9] Joshi R, Ramanarao MV, Baisakh N (2013). *Arabidopsis* plants constitutively overexpressing a *myo*-inositol 1-phosphate synthase gene (*SaINO1*) from the halophyte smooth cordgrass exhibits enhanced level of tolerance to salt stress. Plant Physiol. Biochem..

[10] Kusuda H, Koga W, Kusano M, Oikawa A, Saito K (2015). Ectopic expression of *myo*-inositol 3-phosphate synthase induces a wide range of metabolic changes and confers salt tolerance in rice. Plant Sci..

[11] Majee M, Maitra S, Dastidar KG, Pattnaik S, Chatterjee A, Hait NC, Das KP, Majumder AL (2004). A novel salt-tolerant L-*myo*-Inositol-1-phosphate synthase from *Porteresia coarctata* (Roxb.) tateoka, a halophytic wild rice. molecular cloning, bacterial overexpression, characterization, and functional introgression into tobacco-conferring salt tolerance. J. Biol. Chem..

[12] Patra B, Ray S, Richter A (2010). Enhanced salt tolerance of transgenic tobacco plants by co-expression of *PcINO1* and *McIMT1* is accompanied by increased level of myo-inositol and methylated inositol. Protoplasma.

[13] Tan J, Wang C, Xiang B, Han R, Guo Z (2013). Hydrogen peroxide and nitric oxide mediated cold- and dehydration-induced *myo*-inositol phosphate synthase that confers multiple resistances to abiotic stresses. Plant, Cell Environ..

[14] Wang FB, Zhai H, An YY, Si ZZ, He SZ, Liu QC (2016). Overexpression of *IbMIPS1* gene enhances salt tolerance in transgenic sweetpotato. J. Integr. Agric..

[15] Zhai H, Wang F, Si Z, Huo J, Xing L, An Y, He S, Liu Q (2016). A *myo*-inositol-1-phosphate synthase gene, *IbMIPS1*, enhances salt and drought tolerance and stem nematode resistance in transgenic sweet potato. Plant Biotechnol. J..

[16] Donahue JL, Alford SR, Torabinejad J, Kerwin RE, Nourbakhsh A, Ray WK, Hernick M, Huang X, Lyons BM, Hein PP, Gillaspy GE (2010). The *Arabidopsis thaliana myo*-inositol 1-phosphate synthase1 gene is required for myo-inositol synthesis and suppression of cell death. Plant Cell.

[17] Meng PH, Raynaud C, Tcherkez G, Blanchet S, Massoud K, Domenichini S, Henry Y, Soubigou-Taconnat L, Lelarge-Trouverie C, Saindrenan P, Renou JP, Bergounioux C (2009). Crosstalks between *myo*-inositol metabolism, programmed cell death and basal immunity in *Arabidopsis*. PLoS ONE.

[18] Brearley C, Trethewey RN, Keller R, Brearley CA, Trethewey RN, Mu B (1998). Reduced inositol content and altered morphology in transgenic potato plants inhibited for 1 D-*myo*-inositol 3-phosphate synthase. Plant J..

[19] Nunes ACS, Vianna GR, Cuneo F, Amaya-farfan J, de Capdeville G, Elibio LR (2006). RNAi-mediated silencing of the *myo*-inositol-1-phosphate synthase gene (*GmMIPS1*) in transgenic soybean inhibited seed development and reduced phytate content. Planta.

[20] Khurana N, Chauhan H, Khurana P (2012). Expression analysis of a heat-inducible, *Myo*-inositol-1-phosphate synthase (MIPS) gene from wheat and the alternatively spliced variants of rice and Arabidopsis. Plant Cell Rep..

[21] Supek F, Bosnjak M, Skunca N, Smuc T (2011). REVIGO summarizes and visualizes long lists of gene ontology terms. PLoS ONE.

[22] Kaur H, Shukla RK, Yadav G, Chattopadhyay D, Majee M (2008). Two divergent genes encoding L-*myo*-inositol 1-phosphate synthase1 (CaMIPS1) and 2 (CaMIPS2) are differentially expressed in chickpea. Plant Cell Environ..

[23] Li X-G, Chen S-B, Lu Z-X, Chan T-J, Zeng Q-C, Zu Z (2002). Impact of copy number on transgene expression in tobacco. Acta Bot. Sin..

[24] Fleet JY, Yen CM, Hill EA, Gillaspy GE (2018). Co-suppression of *AtMIPS* demonstrates cooperation of *MIPS1*, *MIPS2* and *MIPS3* in maintaining myo-inositol synthesis. Plant Mol. Biol..

[25] De S (2014). Identification of heat tolerances in wheat at grain filling stage under heat stress environment. Environ. Ecol..

[26] Barnabás B, Jäger K, Fehér A (2008). The effect of drought and heat stress on reproductive. Plant Cell Environ..

[27] Khurana N, Sharma N, Khurana P (2017). Overexpression of a heat stress inducible, wheat *myo*-inositol-1-phosphate synthase 2 (*TaMIPS2*) confers tolerance to various abiotic stresses in *Arabidopsis thaliana*. Agri Gene.

[28] Latrasse D, Jégu T, Meng PH, Mazubert C, Hudik E, Delarue M, Charon C, Crespi M, Hirt H, Raynaud C, Bergounioux C, Benhamed M (2013). Dual function of MIPS1 as a metabolic enzyme and transcriptional regulator. Nucleic Acids Res..

[29] Dreze M (2011). Evidence for network evolution in an Arabidopsis interactome map. Science.

[30] Lorenzo O (2003). Ethylene response FACTOR1 1ntegrates signals from ethylene and jasmonate pathways in plant defense. Plant Cell.

[31] Cao Y-Y, Duan H, Yang L-N, Wang Z-Q, Zhou S-C, Yang J-C (2008). Effect of heat stress during meiosis on grain yield of rice cultivars differing in heat tolerance and its physiological mechanism. Acta Agron. Sin..

[32] Zhang JK, Zong XF, Yu GD, Li JN, Zhang W (2006). Relationship between phytohormones and male sterility in thermo-photo-sensitive genic male sterile (TGMS) wheat. Euphytica.

[33] Marchler-Bauer A (2017). CDD/SPARCLE: Functional classification of proteins via subfamily domain architectures. Nucleic Acids Res..

[34] Nishimura A, Aichi I, Matsuoka M (2007). A protocol for *Agrobacterium*-mediated transformation in rice. Nat. Protoc..

[35] Higo K, Ugawa Y, Iwamoto M, Korenaga T (1999). Plant cis-acting regulatory DNA elements (PLACE) database: 1999. Nucleic Acids Res..

[36] Murray MG, Thompson WF (1980). Rapid isolation of higher weight. DNA..

[37] Sambrook J, Russel DW (2001). Molecular cloning: a laboratory manual.

[38] Singh A, Khurana P (2016). Molecular and functional characterization of a Wheat B2 protein imparting adverse temperature tolerance and influencing plant growth. Front. Plant Sci..

[39] Grabherr MG (2011). Full-length transcriptome assembly from RNA-Seq data without a reference genome. Nat. Biotechnol..

[40] Haas BJ (2013). De novo transcript sequence reconstruction from RNA-seq using the trinity platform for reference generation and analysis. Nat. Protoc..

[41] Robinson MD, McCarthy DJ, Smyth GK (2009). edgeR: A bioconductor package for differential expression analysis of digital gene expression data. Bioinformatics.

[42] Wu Y, Li Q, Chen XZ (2007). Detecting protein-protein interaction by far western blotting. Nat. Protoc..

