# Supplementary material for: Wheat Myo-inositol phosphate synthase influences plant growth and stress responses via ethylene mediated signaling
Source: Sci Rep. 2020 Jul 1;10:10766. doi: 10.1038/s41598-020-67627-w (PMC7329911; doi:10.1038/s41598-020-67627-w)
Supplement: Supplementary file 1 — Supplementary file1 (PDF 315 kb) [file 41598_2020_67627_MOESM1_ESM.pdf]

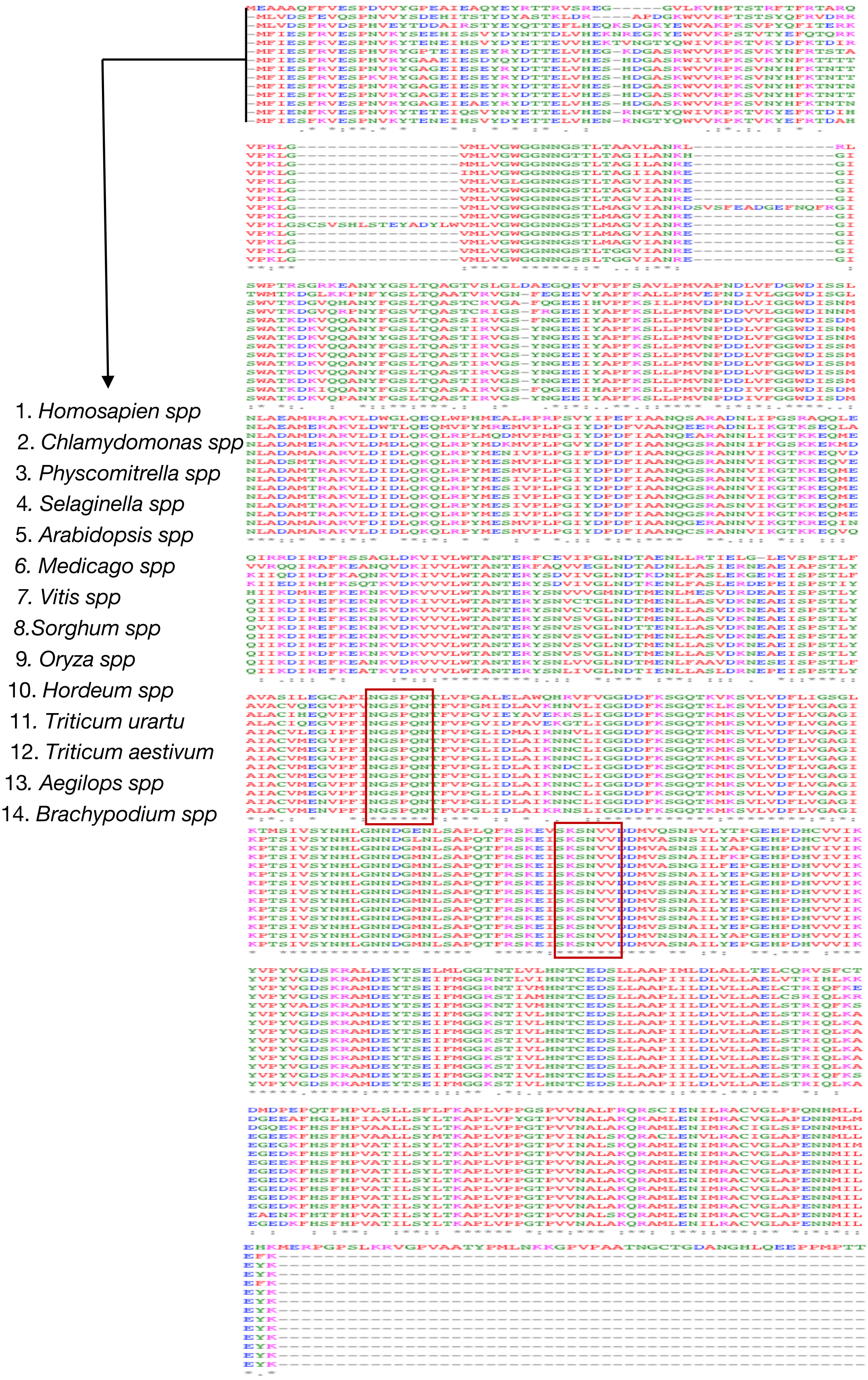

Fig S1. Sequence comparison of *MIPS* from various organism. Sequence were downloaded from ensemble database and fished by HMM search and aligned using clustalOmega. Starred letter shows the identical residue whereas dash shows the gap. Conserved motifs are highlighted by colored lines.

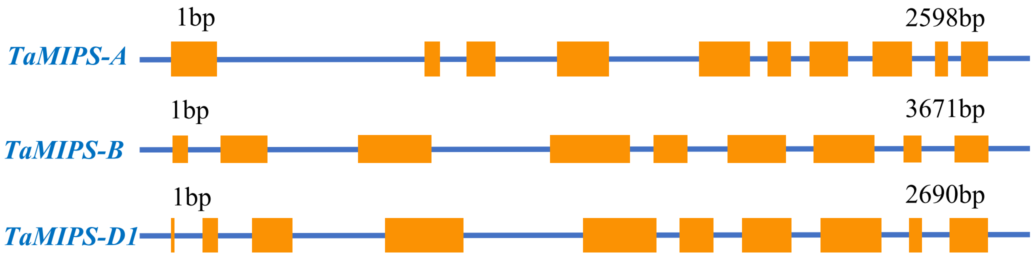

Fig S2. Schematic representation of gene structure of *TaMIPS* homologues. Square box depicts the exons.

Table S1. Molecular information of members of *TaMIPS* gene family.

Gene	Transcript Length (Kb)	Protein Length (a.a.)	Molecular Weight (kDa)	Isoecectric Point (pI)
TaMIPS-A	1341	446	48.75	5.25
TaMIPS-B	1533	510	56.1	5.44
TaMIPS-D	1368	455	49.6	5.25

Table S2. List of enriched cis elements in *TaMIPS-B* promoter.

Element Name	Element Sequence	Biological process involved	Occurrence
ABRELATERD1	ACGTG	cis element involved in ABA responsiveness	7
ABRERATCAL	MACGYGB	cis element involved in ABA responsiveness	5
ACGTATERD1	ACGT	cis element involved in ABA responsiveness	16
CACGTGMOTIF	CACGTG	cis element involved in light responsiveness	6
CGCGBOXAT	VCGCGB	cis element involved in signal-responsiveness	14
CURECORECR	GTAC	cis element involved in copper responsiveness	8
EBOXBNNAPA	CANNTG	cis element present in storage-protein	20
GATABOX	GATA	cis element involved in light responsiveness	8
GCCCORE	GCCGCC	cis element involved in ethylene and jasmonate responsiveness	5
GT1CONSENSUS	GRWAAW	cis element involved in light and salicylic acid responsiveness	5
GTGANTG10	GTGA	cis element in late pollen development genes	16
MYBPZM	CCWACC	cis element involved in red pigmentation of kernel pericarp	5
MYCCONSENSUSAT	CANNTG	cis element involved in ABA responsiveness	20
POLLEN1LELAT52	AGAAA	cis element involved in pollen development genes	5
PRECONSCRHSP70A	SCGAYNRNNNNNNNNNNNNNNNNHD	cis element involved in plastid response	5
SORLIP1AT	GCCAC	cis element involved in light responsiveness	12
SORLIP2AT	GGGCC	cis element involved in light responsiveness	9
